# Umbilical vein remodeling is associated with pregestational maternal overweight

**DOI:** 10.3389/fendo.2024.1483364

**Published:** 2024-12-20

**Authors:** Kamilla Batista da Silva Souza, Luana Caroline Hochberger, Felippe Egon Castrignano Camargo, Gabriely Santos Silva, Giovanna Castrignano Camargo, João Pedro Lourenço Mello, Fernanda Cristina Alcantara Dos Santos, Fernanda Regina Giachini, Núbia de Souza Lobato, Paula Cristina de Souza Souto

**Affiliations:** ^1^ Institute of Health and Biological Science, Federal University of Mato Grosso, Barra do Garças, Brazil; ^2^ Araguaia Multi-User Research Center, Federal University of Mato Grosso, Barra do Garças, Brazil; ^3^ Academic Unit of Health Sciences, Federal University of Jataí, Jataí, Brazil; ^4^ Institute of Health Science, Federal University of Goiás, Goiania, Brazil; ^5^ Red Iberoamericana de Alteraciones Vasculares Asociadas a Transtornos del Embarazo (RIVATREM), Chillán, Chile

**Keywords:** collagen fibers, elastin fibers, glycosaminoglycans, KI-67, umbilical cord

## Abstract

**Introduction:**

Excess weight during pregnancy is a condition that can affect both mother and fetus, through the maternal-fetal interface, which is constituted by the placenta and umbilical cord. The umbilical vein is responsible for transporting oxygen and nutrients to the fetus, and its proper functioning depends on the integrity of its structure. The remodeling of the umbilical vein represents one of the causes of inadequate transport of nutrients to the fetus, being potentially harmful. This study aims to evaluate whether maternal overweight alters the structural characteristics of the umbilical vein.

**Methods:**

Umbilical cords were collected from eutrophic and overweight pregnant women and were processed according to histological routine. We analyzed morphometry parameters, collagen and elastin fibers deposition, glycosaminoglycan level, and cell proliferation.

**Results:**

Veins from overweight pregnant women were found to have greater total area, wall area, wall thickness, and diameter. There was higher collagen labeling in the perivascular region of the overweight group and a higher amount of type III collagen in the vascular smooth muscle. The proliferation of muscle and perivascular cells was higher in overweight pregnant women. A positive, although weak, correlation was observed between BMI and vessel thickness and with type III collagen deposition in vascular smooth muscle.

**Discussion:**

With this study, we show that being overweight can structurally alter the umbilical vein, causing vascular remodeling of the vessel, through hypertrophy and hyperplasia.

## 1 Introduction

Pregestational maternal obesity is associated with unfavorable maternal and fetal outcomes, also favoring the developmental programming of obesity, diabetes, and cardiovascular diseases in early and later life (1, 2). Given the high incidence of obesity affecting pregnancies worldwide, it is imperative to understand the mechanisms involving the deleterious intrauterine environment upon obesity.

The umbilical cord (UC) connects the growing fetus to the placenta, supporting an environment required for a successful pregnancy (3), while it is an easily accessible structure reflecting changes in the intrauterine environment. In this regard, the UC has become an interesting target to investigate, considering its accessibility, almost exclusive fetal derivation, and the influence of maternal and fetal circulation on this component.

The UC circulation is composed of two arteries and a unique vein, all of them surrounded by a mucoid substance known as Wharton’s Jelly (WJ). The umbilical vein carries oxygenated blood and other maternal nutrients to the fetus, while the two umbilical arteries carry low-oxygen blood and fetal metabolites to the placenta (4).

Structurally, the UC vessels display peculiar characteristics, differentiating them from most vessels in the body, since in these vessels, the tunica adventitia is replaced by perivascular cells located around the vessel, known as the perivascular Wharton’s Jelly (PVWJ). This vascular layer has a high density of components including the mesenchymal cells and extracellular matrix components (ECM), standing out type I, III, and V collagen (5), proteoglycans, glycosaminoglycans (GAGs) and elastic fibers (6). This vascular arrangement builds up a strong structure, intending to protect these vessels from compression due to fetal movements and to guarantee the essential flow of substances (7).

Several physiological or pathological conditions may elicit alterations in the vascular architecture, leading to a process known as vascular remodeling. Interestingly, obesity favors vascular remodeling in several vessels (8). The overall mechanisms involved in vascular remodeling include proliferation and differentiation of vascular smooth muscle cells (VSMCs) degradation, disruption of elastic fibers, and ECM component deposition (9, 10).

The present work was designed to evaluate whether pregestational maternal overweight modifies the umbilical vein structure, eliciting its vascular remodeling.

## 2 Materials and methods

### 2.1 Participants

This is a case-control study. Pregnant women were recruited during prenatal visits at UNIMED Hospital from Jataí, Brazil. This study was approved by the Ethical Committee of the Federal University of Jataí and all participants provided written consent. Maternal body composition was assessed between 4–10 weeks of pregnancy. Eligibility criteria included age ≧̸18 years, live newborn delivery; newborn with no fetal malformations on ultrasound and/or postnatal exam, and gestational age ≧̸ 37 weeks. Exclusion criteria included smoking, pre-eclampsia, diabetes, hypertension, artificial reproductive techniques, HIV, syphilis, infections with possible vertical transmission, and deliveries occurring outside the study hospital. The participants were grouped by their pregestational body mass index (BMI) into two groups: control (n=19), where BMI was between 18.5 and 24.9 kg/m²; and overweight (n=16), where BMI was greater than 25.0 kg/m².

### 2.2 Sample collection and processing

The UC samples were collected immediately at delivery, washed in phosphate buffered saline (PBS) and fixed in 4% paraformaldehyde for 24 hours. For histological analysis, the tissues were dehydrated, clarified and embedded in paraffin. Sections with a thickness of 5 μm were adhered to glass slides, deparaffinized, rehydrated and stained for morphometric measurements with hematoxylin-eosin; picrosirius for collagen deposition; resorcin-fuchsin for elastic fibers; alcian blue for glycosaminoglycans precipitation.

The collection of segments near the placental region was standardized due to the direction of fetal blood flow. The images were recorded using a light microscope with a digital camera (Nikon Eclipse E-200, Tokyo, Japan) and software (TCapture). The slides stained with picrosirius were also submitted to polarized light microscopy (Zeiss Axioskop 2). All the images were evaluated using the ImageJ software (National Institutes of Health, Bethesda, Maryland, USA).

### 2.3 Morphometric and histochemical analysis

Vascular measurements were defined as: total vein area (TVA), lumen area (LA), vein wall area (VWA), vein wall thickness (VWT), vein diameter (VD) and wall/lumen ratio. Three images of each vessel were obtained, and the average between them was used as the final value for that sample (40x magnification). For TVA and LA measurements, the contour of the inner and outer vessel wall was evaluated. The subtraction of these measurements (TVA - LA) resulted in the VWA. For VWT, the distance between the outer and inner border of the vessel’s muscular layer was measured in four different regions in the same vessel. The wall/lumen ratio was calculated by a simple division of wall area (mm2) and lumen area (mm2) for each sample. For the VD, two perpendicular measurements of each vein were used. Histochemical analysis of collagen, elastic fibers and glycosaminoglycans was performed by an average of ten images taken of perivascular region and smooth muscle layer (400x magnification). The tool “threshold” was used to set a limit of stain and standardize the protocol. The collagen birefringence analysis was conducted to evaluate type I and type III collagen fibers, according to the hue scale of the image, with values set from 0 to 40 for type I (yellowish red) and 40 to 120 for type III (greenish yellow) (11).

### 2.4 KI-67 immunohistochemistry

Deparaffinized slides were subjected to antigen retrieval with sodium citrate buffer pH 6.0 (#ab64236, Abcam, USA) at a high temperature (95°C) to expose the epitopes. Next, blocking steps were performed with human serum (1:50 in PBS/BSA 1%) and CAS-Block™ (#008120, ThermoFisher, USA). Incubation with a specific KI-67 primary antibody (#ab15580, Abcam, USA, 1:300) was performed at 4°C overnight. Anti-Rabbit IgG biotinylated secondary antibody (#ab6012, Abcam, USA, 1:250) was used. ImmunoCruz^®^ ABC Kit (#sc-516216, Santa Cruz Biotechnology, USA) was used for signal amplification and background reduction, according to the manufacturer’s instructions. Slides were revealed using the ImmPACT™ NovaRED™ Peroxidase (HRP) Substrate kit (Vector Labs, USA). The ImageJ plugin “Cell Counter” was used to count the number of Ki67 positive cells in 10 image fields in the same region, for each sample. Images were deconvoluted using the tool “Color Deconvolution” with “Colour_1” and “Colour_2” used for cell counting and staining intensity, respectively. Then, it was measured the intensity of the staining using the tool “Threshold”, according to the instructions from the “Semi-quantitative determination of protein expression using immunohistochemistry staining and analysis (Crowe and Yue, 2019). The intensity value detected was divided by the number of cells (stained or not) from its respective field, the average was calculated for each sample and presented as percentage (12).

### 2.5 Data analysis

Statistical analysis was performed using GraphPad Prism 8 (Graph Pad Software Inc). Clinical characteristics and histological analysis results were described for the entire sample and represented by mean ± SEM. The distribution of the variables was tested using the Shapiro-Wilk test. Correlation analysis was performed between the variables using Pearson’s linear coefficient. Differences between groups were compared using the *Student* t-test (parametric data) or Mann-Whitney test (non-parametric data) and values were considered statistically significant when p < 0.05.

## 3 Results

The current study comprises 35 pregnant women, who attended the inclusion and exclusion criteria, divided into control (n=19) and overweight group (n=16). The mean age among mothers and gestational age were similar between groups. BMI was reduced in control compared to the overweight group (p<0.0001). The newborn’s weight and the maternal weight gain during the pregnancy were similar between groups (**Table 1**).

**Table 1:** General data in the groups.

Parameter	Groups	Mean ± SEM	P value
**Age (years)**	Control Overweight	28.83 ± 1.34 30.06 ± 1.31	0.516
**Gestational Age (weeks)**	Control Overweight	38.62 ± 0.14 38.92 ± 0.19	0.214
**BMI (kg/m²)**	Control Overweight	22.27 ± 0.44 28.02 ± 0.71	< 0.0001*
**Newborn weight (kg)**	Control Overweight	3.26 ± 0.15 3.45 ± 0.08	0.273
**Maternal weight gain (kg)**	Control Overweight	12.31 ± 0.72 12.48 ± 0.98	0.889

The difference between groups was assessed using the Mann-Whitney test. *p<0.05 vs. control. BMI, body mass index; SEM, standard error of the mean. Bold values indicate p<0.05.

### 3.1 Morphometric measurements of umbilical vein

**Figure 1:**
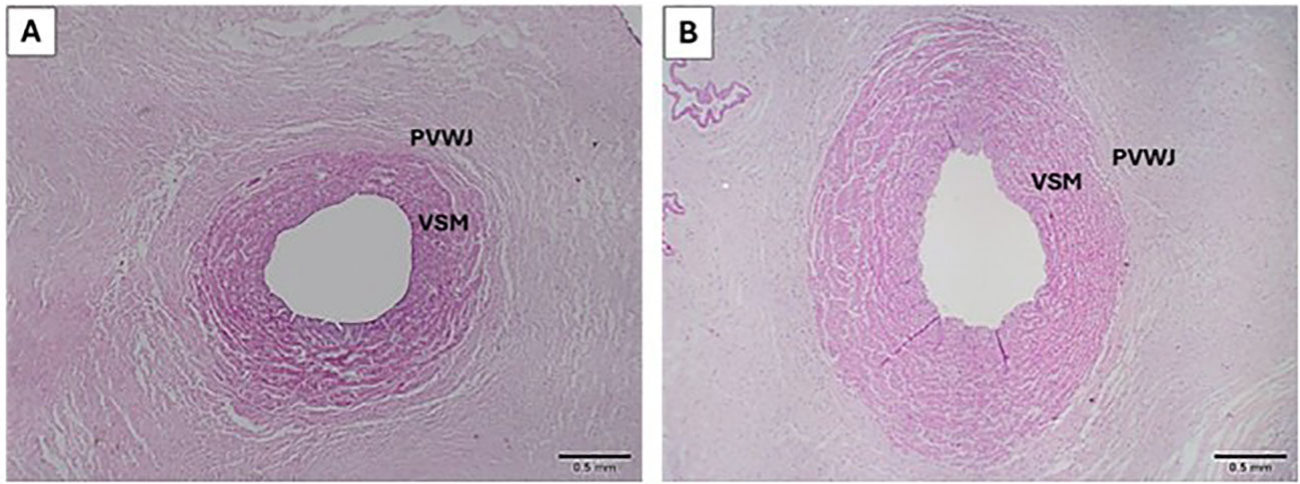
Representatives images of umbilical veins from control **(A)** and overweight **(B)** groups. PVWJ, *Perivascular Wharton's Jelly*; VSM, Vascular Smooth Muscle. *H&E 40x*.

Morphometric measurements are shown in **Table 2** and the umbilical veins from groups are represented in **Figure 1**. Umbilical vein morphometric analysis revealed a significant increase in total area, vein wall area, diameter and thickness in the overweight group, compared to the control. The total lumen area, the wall/lumen ratio and diameter did not show significant differences when the two groups were compared between the groups.

**Table 2:** Values of umbilical cord vein morphometry analysis of control and overweight pregnant women.

Parameter	Groups	Mean ± SEM	P value
**Total area (mm²)**	Control Overweight	3.10 ± 0.22 4.16 ± 0.45	0.04 *
**Lumen areal (mm²)**	Control Overweight	0.71 ± 0.11 0.86 ± 0.13	0.385
**Wall areal (mm²)**	Control Overweight	2.43 ± 0.17 3.29 ± 0.36	0.04 *
**Wall/lumen area**	Control Overweight	4.02 ± 0.37 4.40 ± 0.45	0.519
**Wall thickness (mm)**	Control Overweight	0.48 ± 0.02 0.58 ± 0.04	0.035 *
**Diameter (mm)**	Control Overweight	1.98 ± 0.07 2.31 ± 0.13	0.034 *

The difference between groups was assessed using the Mann-Whitney test. *p<0.05 vs. control. SEM, standard error of the mean. Bold values indicate p<0.05.

### 3.2 Collagen and elastin deposition

**Figure 2:**
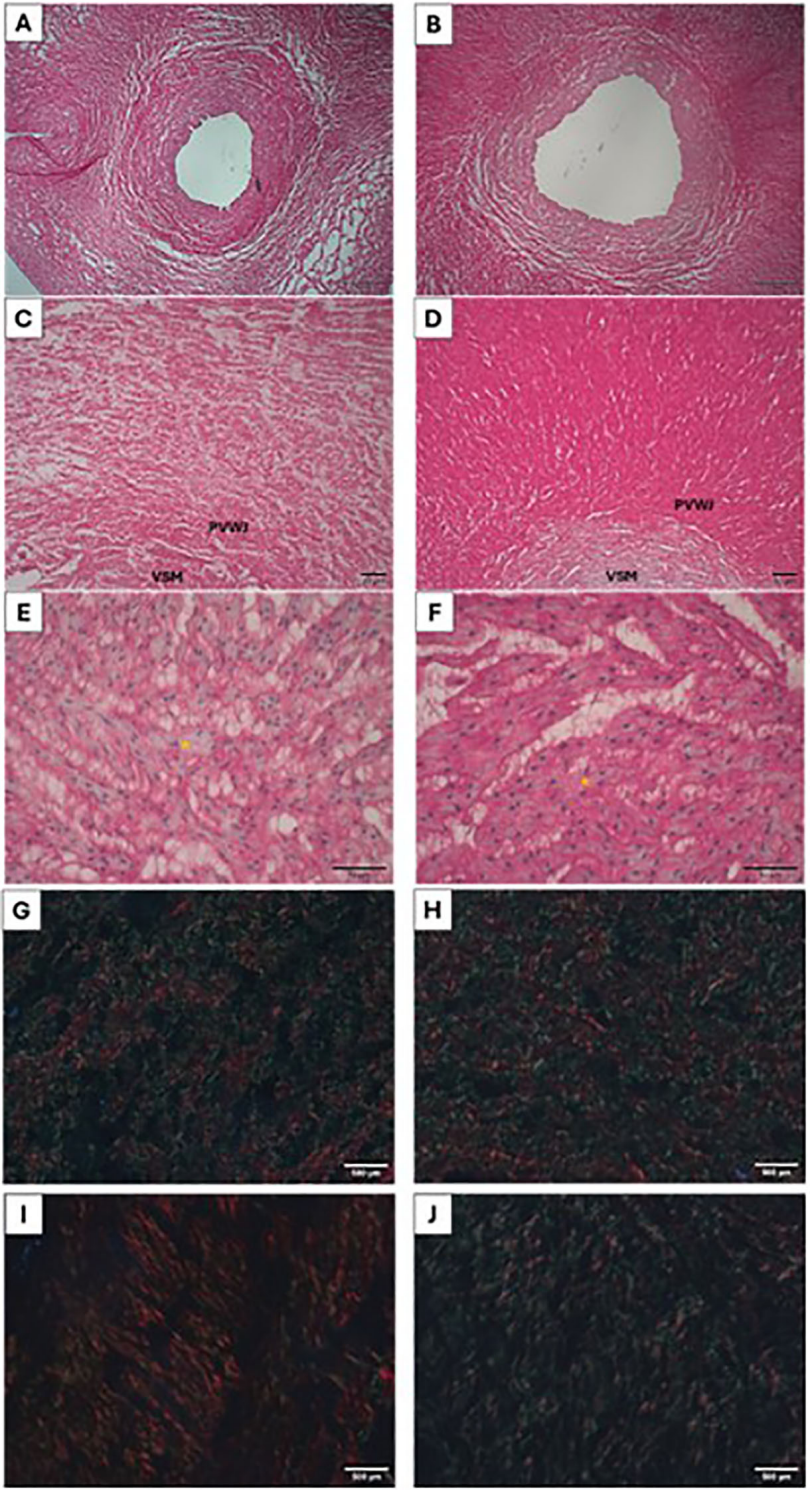
Micrograph of the umbilical vein for collagen analysis in the control **(A, C, E, G, I)** and overweight **(B, D, F, H, J)** groups. In **(A, C)**, it is possible to observe less collagen deposition (red) in the perivascular region when compared to **(B, D)** (Picrosirius 40x and 100x). Collagen deposition between smooth muscle cells (red), nuclei stained with hematoxylin (yellow asterisks) [**(E, F)**, (Picrosirius 400x)]. Type I (red) and type III (green) collagen deposition in the perivascular **(G, H)** and vascular smooth cells **(I, J)** regions, under polarized light (Picrosirius 200x).

Total collagen and elastin deposition was evaluated around the PVWJ and between the vascular smooth muscle (VSM) cells from the umbilical vein. Greater deposition of total collagen in the PVWJ from umbilical veins was observed in the overweight group, compared to the control. However, total collagen deposition between the VSM was similar between groups (**Figure 2**). Polarized microscopy was performed to access type I and III collagen quantification. In the PVWJ region, type I and III collagens were similar between groups. Interestingly, collagen III was more abundant in VSM in the umbilical veins from the overweight group, compared to the control, and no differences were observed related to type I collagen in VSM between groups (**Table 3**, **Figure 2**).

**Table 3:** Collagen deposition in the perivascular Wharton’s Jelly and around Smooth Muscle Cells.

Parameter	Groups	Mean ± SEM	P value
**Total collagen PVWJ** **(% area)**	Control Overweight	57.74 ± 0.02 71.08 ± 0.50	<0.0001 *
**Total collagen VSM** **(% area)**	Control Overweight	13.74 ± 1.39 17.13 ± 2.12	0.192
**Collagen I PVWJ** **(% area)**	Control Overweight	14.89 ± 2.05 12.66 ± 2.64	0.509
**Collagen III PVWJ** **(% area)**	Control Overweight	6.32 ± 1.41 9.43 ± 1.91	0.200
**Collagen I VSM** **(% area)**	Control Overweight	6.87 ± 1.36 4.34 ± 0.82	0.122
**Collagen III VSM** **(% area)**	Control Overweight	3.48 ± 0.50 5.14 ± 0.54	0.031 *

The difference between groups was assessed using the Mann-Whitney test. *p<0.05 vs. control. PVWJ, Perivascular Wharton’s Jelly; SEM, standard error of the mean; VSM, Vascular Smooth Muscle. Bold values indicate p<0.05.

**Figure 3:**
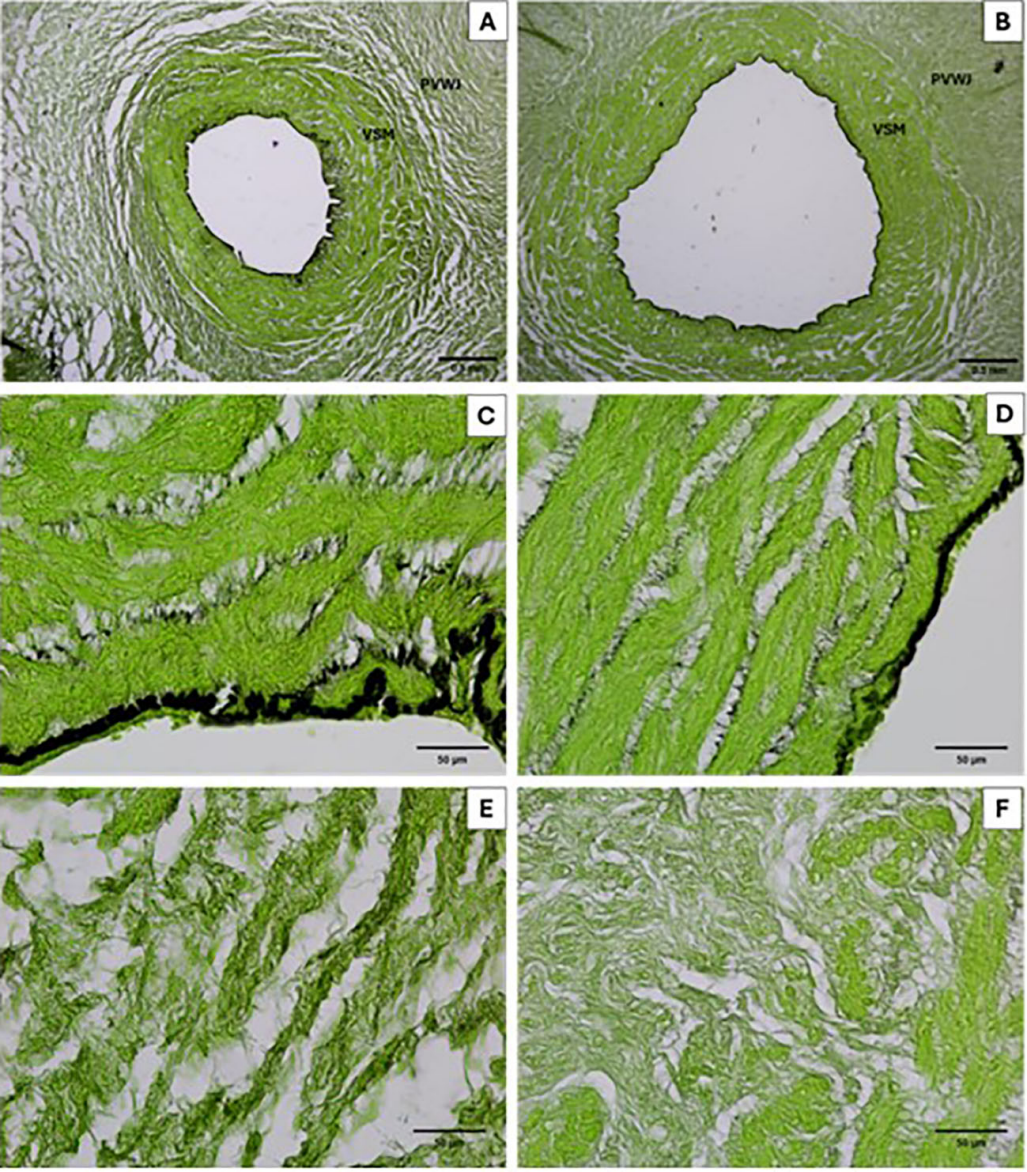
Micrograph of the umbilical vein for elastin analysis in the control **(A, C, E)** and overweight **(B, D, F)** groups. In **(A, C)**, it is possible to observe more elastin deposition (black) in the vascular region when compared to **(B, D)** (Resorcin-fuchsin 40x and 400x). No difference were observed in the perivascular region **(E, F)**.

Elastin deposition was smaller between VSM from the overweight group, compared to the control. No differences were observed in elastin deposition around PVWJ from the umbilical vein (**Table 4**, **Figure 3**).

**Table 4:** Elastin deposition in the perivascular Wharton’s Jelly and around Smooth Muscle Cells.

Parameter	Groups	Mean ± SEM	P value
**Elastin PVWJ** **(% area)**	Control Overweight	4.94 ± 0.90 3.93 ± 0.67	0.375
**Elastin VSM** **(% da area)**	Control Overweight	0.71 ± 0.25 0.15 ± 0.07	0.043 *

The difference between groups was assessed using the Mann-Whitney test. *p<0.05 vs. control. PVWJ, Perivascular Wharton’s Jelly; SEM, standard error of the mean; VSM, Vascular Smooth Muscle. Bold values indicate p<0.05.

### 3.3 Glycosaminoglycans deposition

**Figure 4:**
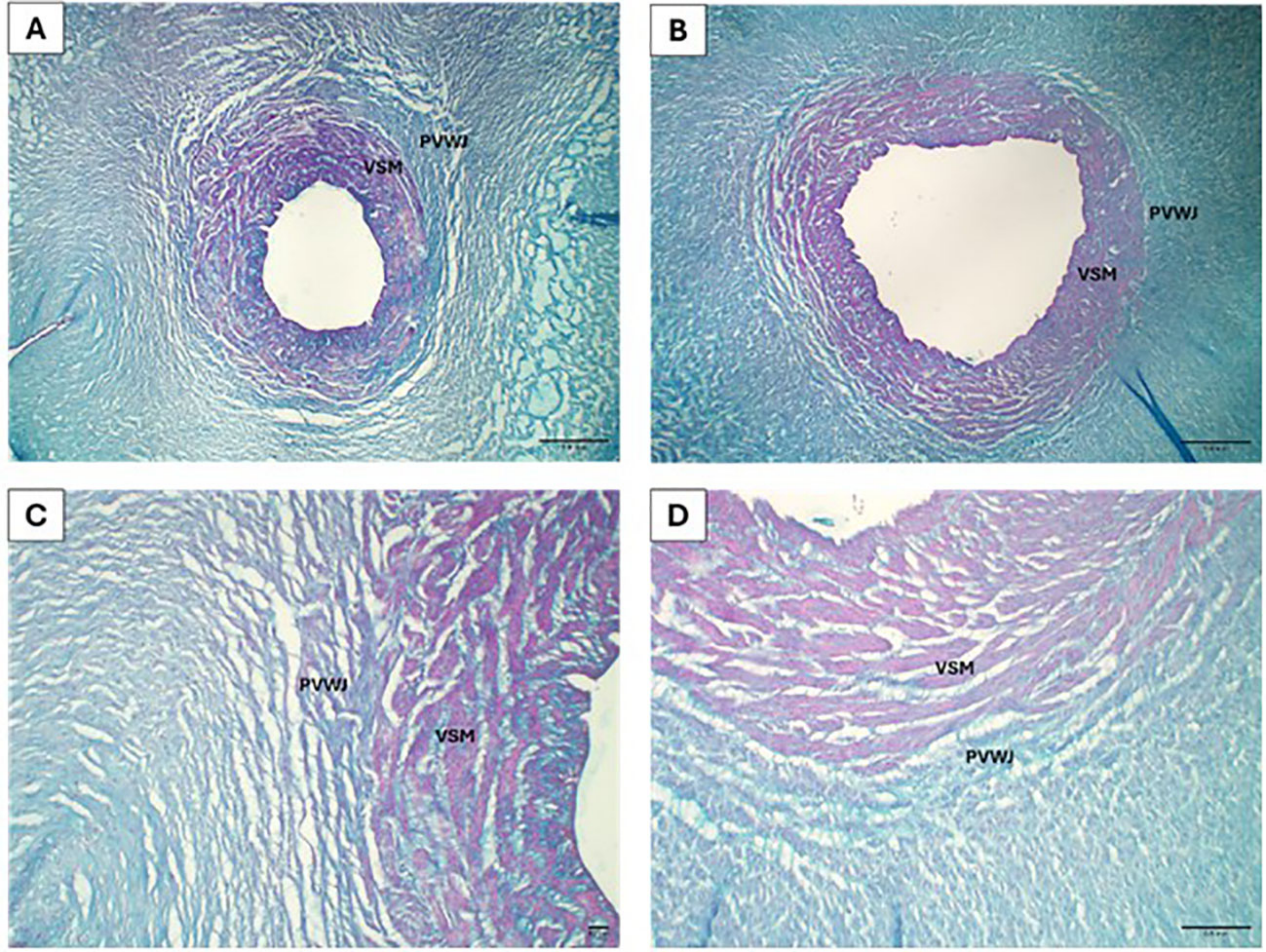
Micrograph of the umbilical vein for glycosaminoglycans analysis in the control **(A, C)** and overweight **(B, D)** groups. Veins from the control **(A)** and overweight **(B)** groups are observed, with the glycosaminoglycans stained in blue (Alcian blue, 40x). The delimitation between PVWJ and VSM is evident in the control **(C)** and overweight **(D)** groups (Alcian blue, 100x).

The distribution of GAGs was evaluated in the perivascular and smooth muscle regions of the umbilical vein (**Figure 4**). There was no difference in GAGs deposition between the groups, as shown in **Table 5**.

**Table 5:** Glycosaminoglycans deposition in the perivascular Wharton’s Jelly and around Smooth Muscle Cells.

Parameter	Groups	Mean ± SEM	P value
**Alcian Blue PVGJ** **(% area)**	Control Overweight	56.32 ± 7.38 54.57 ± 10.12	0.921
**Alcian Blue VSM** **(% area)**	Control Overweight	30.25 ± 3.98 22.65 ± 4.73	0.228

The difference between groups was assessed using the Mann-Whitney test. PVWJ, Perivascular Wharton’s Jelly*;* SEM, standard error of the mean; VSM, Vascular Smooth Muscle. Bold values indicate p<0.05.

### 3.4 Cell proliferation

**Figure 5:**
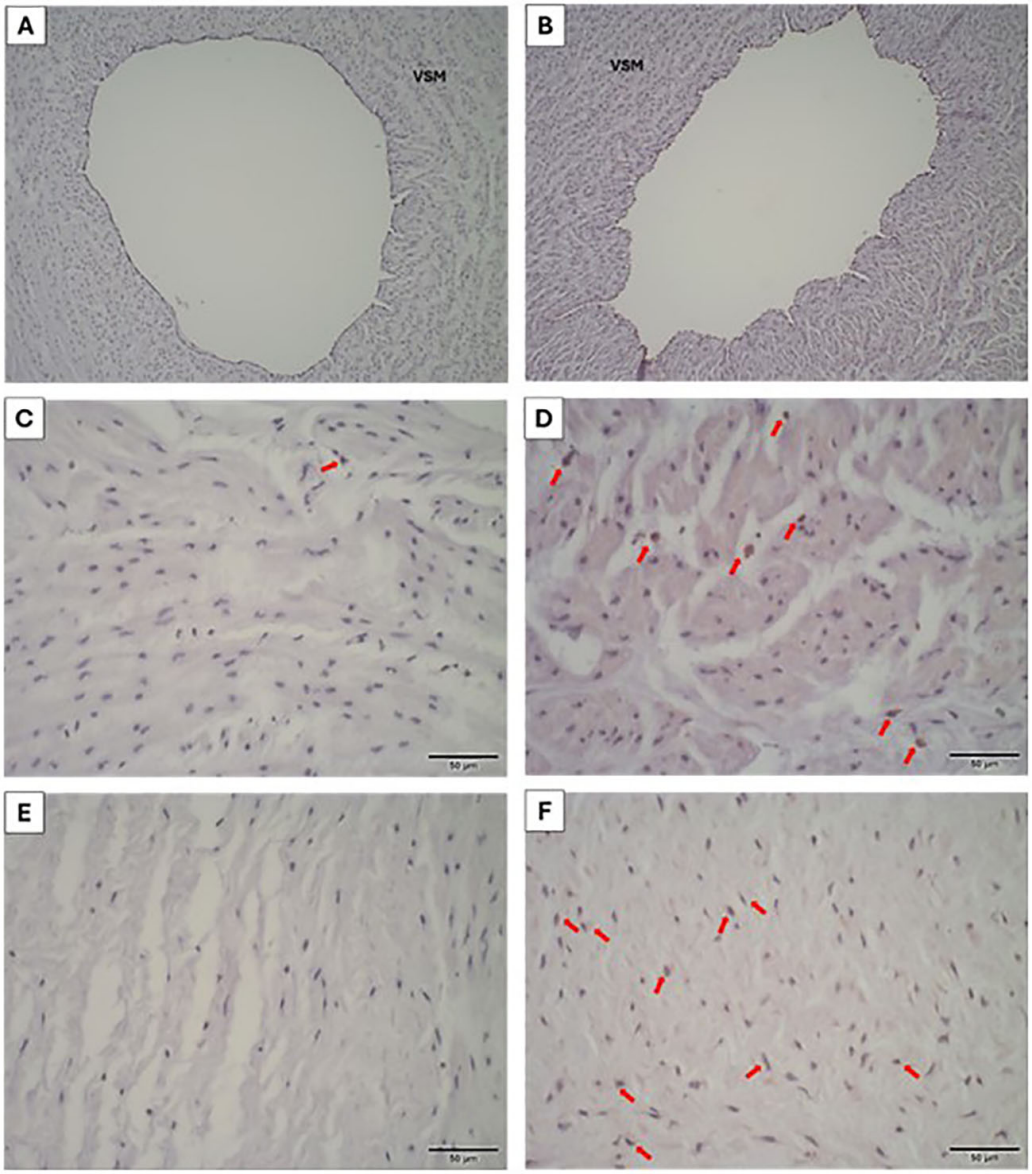
Cell proliferation in the umbilical vein from control **(A, C, E)** and overweight **(B, D, F)** groups. Positive staining (red arrows) for KI-67 in the VSM **(C, D)** and PVWJ **(E, F)** regions (IHC, 400x).

Cell proliferation, accessed with positive labeling for the KI-67 antibody, was observed in the PVWJ and VSM from umbilical veins. In the overweight group, both regions showed a higher percentage of labeled cells when compared to the control group (**Table 6**), indicating greater cell proliferation in these regions in the group (**Figure 5**).

**Table 6:** Cell proliferation in umbilical veins from control and overweight groups.

Parameter	Groups	Mean ± SEM	P value
**PVWJ** **(% stained cells)**	Control Overweight	0.07 ± 0.02 1.72 ± 0.54	0.008 *
**VSM** **(% stained cells)**	Control Overweight	0.14 ± 0.04 0.38 ± 0.06	0.002 *

The difference between groups was assessed using the Mann-Whitney test. *p<0.05 vs. control. PVWJ, Perivascular Wharton’s Jelly*;* SEM, standard error of the mean; VSM, Vascular Smooth Muscle. Bold values indicate p<0.05.

### 3.5 BMI and fetal weight correlation

The correlation test showed a positive association between BMI and vein thickness (r=0.357; p=0.035) and type III collagen in vascular smooth muscle (r=0.567; p=0.022), as shown in **Figure 2** and **Table 7**. Fetal weight negatively correlated with the lumen area (r=0.4038; p=0.027) and positively correlated with the wall/lumen area (r=0.4050; p=0.023), **Table 8**. For those other parameters, no significant correlations were found.

**Table 7:** Correlations values between BMI and umbilical vein histological parameters from the control and overweight groups.

Correlation	Spearman (r)	P Value
**BMI vs. Total area (UV)**	0.1999	0.249
**BMI vs. Lumen area (UV)**	0.01952	0.911
**BMI vs. Wall thickness (UV)**	0.2501	0.147
**BMI vs. Ratio wall/lumen (UV)**	0.1638	0.347
**BMI vs. Thickness (UV)**	0.3571	0.035 *
**BMI vs. Diameter (UV)**	0.2473	0.152
**BMI vs. Picrosirius PVWJ**	0.4072	0.054
**BMI vs. Picrosirius VSM**	0.3509	0.101
**BMI vs. Type I Collagen PVWJ**	-0.3134	0.237
**BMI vs. Type III Collagen PVWJ**	0.4604	0.073
**BMI vs. Type I Collagen VSM**	-0.1336	0.954
**BMI vs. Type III Collagen MLV**	0.5674	0.022 *
**BMI vs. GAGs PVWJ**	0.1189	0.547
**BMI vs. GAGx VSM**	0.01139	0.622

The difference between groups was assessed using the Mann-Whitney test. *p<0.05 vs. control. PVWJ, Perivascular Wharton’s Jelly*;* r, Spearman correlation coefficient; UV, Umbilical Vein; VSM, Vascular Smooth Muscle. Bold values indicate p<0.05.

**Table 8:** Correlations values between fetal weight and umbilical vein histological parameters from the control and overweight groups.

Parameter	Spearman (r)	P value
**Fetal weight x Total area (mm²)**	-0.1785	0.336
**Fetal weight x Lumen area (mm²)**	-0.4038	0.027*
**Fetal weight x Wall thickness (mm²)**	-0.1218	0.513
**Fetal weight x Wall/lumen area**	0.4050	0.023*
**Fetal weight x Wall thickness (mm)**	-0.0781	0.716
**Fetal weight x Diameter (mm)**	-0.1892	0.308

The difference between groups was assessed using the Mann-Whitney test. *p<0.05 vs. control. r, Sperman correlation coefficient; UV, Umbilical Vein. Bold values indicate p<0.05.

## 4 Discussion

In the present study, it was demonstrated that the umbilical vein undergoes extensive remodeling in overweight women, increasing the caliber of this vessel through hypertrophy and hyperplasia. Furthermore, greater collagen deposition was observed in the perivascular region, with a predominance of type III collagen in the smooth muscle region, but without changes in glycosaminoglycan deposition. Additionally, a correlation between BMI and vein thickness and type III collagen in smooth muscle was established.

The umbilical vein plays a crucial role during pregnancy, being responsible for transporting blood rich in oxygen and nutrients from the placenta to the fetus (13). This vein connects the placenta to the fetal liver, where blood is distributed throughout the fetal body through fetal circulation. This ensures that the fetus receives the nutrients and oxygen necessary for proper development (14). The blood flow in the umbilical vein is influenced by the helical pattern of the umbilical vessels (15) with an internal pressure of around 2 to 9 mmHg, with an acceleration of 10-22 cm/s. Under normal conditions, the vascular wall responds to shear stress exerted by the blood through the production of vasoactive factors and cytokines (16).

Vascular remodeling occurs as a body response to different stimuli, both physiological and pathological. This process involves changes in the structure and function of blood vessels, such as arteries and veins, and can occur because of the maternal environment, including high blood pressure (17), hyperglycemia (18) and obesity. The vascular remodeling contributes to congenital malformations and reproductive losses, but the mechanisms related to these associations are still unclear (19).

Vascular smooth muscle cells alteration is related to vascular remodeling, since they may switch from contractile to synthetic phenotype (20). This plasticity is necessary to adapt to the different conditions of the vascular tissue upon signs of stress or injury (21). Recent studies have demonstrated that the synthetic phenotype is complex and may acquire characteristics of different mesenchymal lineages, including osteoblastic, chondrocyte and adipocytic (22). This implies that local factors present in the pathological environment probably drive part of this inadequate differentiation. The phenotypic switching contributes to changes in the primary function of these cells from regulation of vessel tone, blood pressure, and blood flow through cells focused on producing extracellular matrix components, assuming a proliferative and migratory phenotype (23).

In pregnancies complicated by obesity, there may be dysregulation in proliferative control in umbilical cord cells. The percentage of cells positive for the cell proliferation marker, KI-67, was higher in the overweight group. In addition to the association with increased vessel caliber resulting from the need to adapt to the adverse environment, factors that are elevated in obesity, such as leptin, induce the proliferation of VSM cells, using the nuclear factor kappa B (NF-kB) and kinase pathways. regulated by extracellular signals (ERK) 1/2 (24). Hence, PVWJ displays some paracrine actions, and releases several substances including growth factors, cytokines, chemokines, exosomes, and microparticles (25), and in theory, these molecules may directly modulate VSM cell proliferation (26). This may occur even due to the interaction of endothelium and PVWJ, since it was demonstrated that the secretome of human endothelial cell-differentiated mesenchymal stem cell enhanced proliferation and wound healing *in vivo*, in a type-2 diabetes model (27). It was shown that maternal obesity negatively affects PVWJ, suggesting that epigenetic mechanisms may impact PVWJ mesenchymal stromal/stem cells since the methylation of CpG sites was enhanced in samples from obese women (27). However, new, more in-depth studies are necessary to highlight the molecular mechanisms involved in the VSM cell proliferation observed in the umbilical vein.

The presence of arterial stiffness is commonly reported during obesity, both in humans (28–30) and in murine studies (31, 32). The vascular mechanical properties are dependent on the quantity and organization of extracellular matrix components, mainly in relation to collagen and elastin (33).

Collagen is crucial to the normal function and longevity of blood vessels, providing the structure and strength necessary to withstand the demands of the circulatory system (34). Total collagen deposition was increased in the perivascular region of the umbilical vein, indicating a response to structural changes resulting from obesity, in addition to an increase in the proliferation of cells in this region. These findings are consistent with the literature, in which changes in vessel conformation in response to stress signals increase the deposition of extracellular matrix components and promote cell proliferation (35). Furthermore, type III collagen is increased in the VSM of the umbilical vein from overweight pregnant women. A similar observation was done in the umbilical vein of pregnant women with pre-eclampsia (36). Type III collagen has different characteristics from type I, as it does not have high density, its fibers are thinner and have less resistance, allowing the tissue to undergo greater distension (37). In this sense, we can infer that vascular adaptation occurs in the face of remodeling induced by excess weight, in the same way that occurs in the brachial arteries of overweight individuals (38).

A comparison between the saphenous vein and the umbilical vein showed that the first one is stiffer, considering a greater amount of elastin and collagen (39). With this information in mind, the umbilical vein from the control group showed greater stiffness than the overweight group. This fact impacts on the capacity of the vessel to influence the blood flow. In other conditions such as preeclampsia, reduced elastin expression was observed in umbilical cord veins (40). Defective elastin is associated with disorganized elastic fiber deposition and VSM proliferation, causing arterial stiffening (41, 42).

Another extracellular matrix parameter analyzed was the glycosaminoglycans distribution. These molecules form an important network for the diffusion of substances, due to their hydrophilic characteristic, facilitating vessel nutrition (43). However, these polysaccharides also have an important effect on vascular mechanics, contributing to the elastic capacity of the tissue (44). In our data, there were no differences in the number of GAGs in the vascular wall or extracellular matrix. Analysis of the umbilical vein from preeclamptic women displayed similar results (45).

The alterations mentioned above may contribute to several undesirable scenarios regarding fetal health. For example, intrauterine growth restriction may occur due to decreased umbilical vein capacity to provide oxygen and nutrients. Yet, reduced nutritional/oxygen apport elicits commitment to organ development. Yet, fibrosis can increase the risk of hypoxia in the fetus, resulting in perinatal asphyxia, and impacting neurological development (46). Another complication due to low oxygenation and undernutrition is fetal stress, a condition that may be monitored during prenatal examination, leading to interventions that include early birth (47).

Furthermore, this study has limitations associated with the number of samples and collection origin, requiring a larger cohort, with a delimitation between overweight and obesity to infer the influences of each degree separately. Furthermore, here the components of the extracellular matrix were analyzed separately, and tests that demonstrate the interaction dynamics of these components can be used to better understand these findings.

In summary, our data show that maternal excess weight structurally alters the umbilical cord vessels, opening a range of possibilities to be explored in the field of umbilical cord histopathology associated with excess weight. Our findings have clinical relevance, as the context of obesity is common among pregnant women (48).

## Data Availability

The raw data supporting the conclusions of this article will be made available by the authors, without undue reservation.

## References

[1] Strauss A. Obesity in pregnant women: maternal, fetal, and transgenerational consequences. Eur J Clin Nutr. (2021) 75:1681–3. doi: 10.1038/s41430-021-01015-z PMC8636246 34702963

[2] Wilson RM Marshall NE Jeske DR Purnell JQ Thornburg K Messaoudi I. Maternal obesity alters immune cell frequencies and responses in umbilical cord blood samples. Pediatr Allergy Immunol. (2015) 26:344–51. doi: 10.1111/pai.2015.26.issue-4 PMC9271931 25858482

[3] Di Naro E Ghezzi F Raio L Franchi M D’Addario V. Umbilical cord morphology and pregnancy outcome. Eur J Obstetrics Gynecology Reprod Biol. (2001) 96:150–7. doi: 10.1016/S0301-2115(00)00470-X 11384798

[4] Spurway J Logan P Pak S. The development, structure and blood flow within the umbilical cord with particular reference to the venous system. Australas J Ultrasound Med. (2012) 15:97–102. doi: 10.1002/j.2205-0140.2012.tb00013.x 28191152 PMC5025097

[5] Davies JE Walker JT Keating A. Concise review: Wharton’s Jelly: the rich, but enigmatic, source of mesenchymal stromal cells. Stem Cells Transl Med. (2017) 6:1620–30. doi: 10.1002/sctm.16-0492 PMC5689772 28488282

[6] Sobolewski K Bańkowski E Chyczewski L Jaworski S. Collagen and glycosaminoglycans of Wharton’s Jelly. Neonatology. (1997) 71:11–21. doi: 10.1159/000244392 8996653

[7] Kliman HJ. The umbilical cord. Encyclopedia Reprod. (2006).

[8] Melo LG Gnecchi M Ward CA Dzau VJ. Vascular remodeling in health and disease. In: Cardiovascular Medicine. Springer London, London (2007). p. 1541–65.

[9] Martínez-Martínez E Miana M Jurado-López R Bartolomé MV Souza Neto FV Salaices M. The potential role of leptin in the vascular remodeling associated with obesity. Int J Obes. (2014) 38:1565–72. doi: 10.1038/ijo.2014.37 24583853

[10] van Varik BJ Rennenberg RJMW Reutelingsperger CP Kroon AA de Leeuw PW Schurgers LJ. Mechanisms of arterial remodeling: lessons from genetic diseases. Front Genet. (2012) 3. doi: 10.3389/fgene.2012.00290 PMC3521155 23248645

[11] Bedoya SAO Conceição LG Viloria MIV Loures FH Valente FL Amorim RL. Caracterização de colágenos tipos I e III no estroma do carcinoma de células escamosas cutâneo em cães. Arq Bras Med Vet Zootec. (2016) 68:147–54. doi: 10.1590/1678-4162-8484

[12] Crowe A Yue W. Semi-quantitative determination of protein expression using immunohistochemistry staining and analysis: an integrated protocol. Bio Protoc. (2019) 9. doi: 10.21769/BioProtoc.3465 PMC6924920 31867411

[13] Sánchez-Trujillo L García-Montero C Fraile-Martinez O Guijarro LG Bravo C De Leon-Luis JA. Considering the effects and maternofoetal implications of vascular disorders and the umbilical cord. Medicina (B Aires). (2022) 58:1754. doi: 10.3390/medicina58121754 PMC9782481 36556956

[14] Barbieri M Di Martino DD Ferrazzi EM Stampalija T. Umbilical vein blood flow: State-of-the-art. J Clin Ultrasound. (2023) 51:318–25. doi: 10.1002/jcu.23412 36785504

[15] de Laat M Franx A van Alderen E Nikkels P Visser G. The umbilical coiling index, a review of the literature. J Maternal-Fetal Neonatal Med. (2005) 17:93–100. doi: 10.1080/jmf.17.2.93.100 16076615

[16] Pennati G. Biomechanical properties of the human umbilical cord. Biorheology. (2001) 38:355–66. 12016319

[17] Lan Y Yang Z Huang M Cui Z Qi Y Niu H. Morphological and structural changes of umbilical veins and clinical significance in preeclampsia. Hypertens Pregnancy. (2018) 37:105–10. doi: 10.1080/10641955.2017.1420799 29733777

[18] Tenaw Goshu B. Histopathologic impacts of diabetes mellitus on umbilical cord during pregnancy. Pediatr Health Med Ther. (2022) 13:37–41. doi: 10.2147/PHMT.S323812 PMC8863384 35210900

[19] Ennazhiyil SV Ramakrishnan P Akshara V Premlal K Chitra S Benjamin W. Effects of gestational diabetes mellitus on umbilical cord morphology: A comparative study. J Clin Diagn Res. (2019) 13:AC01–04. doi: 10.7860/JCDR/2019/40085.12543

[20] Xin Y Zhang Z Lv S Xu S Liu A Li H. Elucidating VSMC phenotypic transition mechanisms to bridge insights into cardiovascular disease implications. Front Cardiovasc Med. (2024) 11. doi: 10.3389/fcvm.2024.1400780 PMC11128571 38803664

[21] Shi J Yang Y Cheng A Xu G He F. Metabolism of vascular smooth muscle cells in vascular diseases. Am J Physiology-Heart Circulatory Physiol. (2020) 319:H613–31. doi: 10.1152/ajpheart.00220.2020 32762559

[22] Elmarasi M Elmakaty I Elsayed B Elsayed A Al Zein J Boudaka A. Phenotypic switching of vascular smooth muscle cells in atherosclerosis, hypertension, and aortic dissection. J Cell Physiol. (2024) 239. doi: 10.1002/jcp.v239.4 38291732

[23] Frismantiene A Philippova M Erne P Resink TJ. Smooth muscle cell-driven vascular diseases and molecular mechanisms of VSMC plasticity. Cell Signal. (2018) 52:48–64. doi: 10.1016/j.cellsig.2018.08.019 30172025

[24] Huang F Xiong X Wang H You S Zeng H. Leptin-induced vascular smooth muscle cell proliferation via regulating cell cycle, activating ERK1/2 and NF- B. Acta Biochim Biophys Sin (Shanghai). (2010) 42:325–31. doi: 10.1093/abbs/gmq025 20458445

[25] KL P Kandoi S Misra R V S R K Verma RS. The mesenchymal stem cell secretome: A new paradigm towards cell-free therapeutic mode in regenerative medicine. Cytokine Growth Factor Rev. (2019) 46:1–9. doi: 10.1016/j.cytogfr.2019.04.002 30954374

[26] Wang Q Yang Q Wang Z Tong H Ma L Zhang Y. Comparative analysis of human mesenchymal stem cells from fetal-bone marrow, adipose tissue, and Warton’s jelly as sources of cell immunomodulatory therapy. Hum Vaccin Immunother. (2016) 12:85–96. doi: 10.1080/21645515.2015.1030549 26186552 PMC4962749

[27] Ormazabal V Nova-Lampeti E Rojas D Zúñiga FA Escudero C Lagos P. Secretome from human mesenchymal stem cells-derived endothelial cells promotes wound healing in a type-2 diabetes mouse model. Int J Mol Sci. (2022) 23. doi: 10.3390/ijms23020941 PMC8779848 35055129

[28] Kim HL Ahn DW Kim SH Lee DS Yoon SH Zo JH. Association between body fat parameters and arterial stiffness. Sci Rep. (2021) 11:20536. doi: 10.1038/s41598-021-00175-z 34654852 PMC8519992

[29] Safar ME Czernichow S Blacher J. Obesity, arterial stiffness, and cardiovascular risk. J Am Soc Nephrology. (2006) 17:S109–11. doi: 10.1681/ASN.2005121321 16565231

[30] Zebekakis PE Nawrot T Thijs L Balkestein EJ van der Heijden-Spek J Van Bortel LM. Obesity is associated with increased arterial stiffness from adolescence until old age. J Hypertens. (2005) 23:1839–46. doi: 10.1097/01.hjh.0000179511.93889.e9 16148607

[31] Gil-Ortega M Martín-Ramos M Arribas SM González MC Aránguez I Ruiz-Gayo M. Arterial stiffness is associated with adipokine dysregulation in non-hypertensive obese mice. Vascul Pharmacol. (2016) 77:38–47. doi: 10.1016/j.vph.2015.05.012 26028606

[32] Gkousioudi A Yu X Ferruzzi J Qian J Wainford RD Seta F. Biomechanical properties of mouse carotid arteries with diet-induced metabolic syndrome and aging. Front Bioeng Biotechnol. (2022) 10. doi: 10.3389/fbioe.2022.862996 PMC8980683 35392404

[33] Aroor AR Jia G Sowers JR. Cellular mechanisms underlying obesity-induced arterial stiffness. Am J Physiology-Regulatory Integr Comp Physiol. (2018) 14:R387–98. doi: 10.1152/ajpregu.00235.2016 PMC5899249 29167167

[34] Halper J. Basic components of vascular connective tissue and extracellular matrix. Adv Pharmacol. (2018) 81:95–127. doi: 10.1016/bs.apha.2017.08.012 29310805

[35] Xu J Shi GP. Vascular wall extracellular matrix proteins and vascular diseases. Biochim Biophys Acta (BBA) - Mol Basis Disease. (2014) 1842:2106–19. doi: 10.1016/j.bbadis.2014.07.008 PMC4188798 25045854

[36] Romanowicz L Jaworski S. Collagen of umbilical cord vein and its alterations in pre-eclampsia. Acta Biochim Pol. (2002) 49:451–8. doi: 10.18388/abp.2002_3804 12362987

[37] Wittig C Szulcek R. Extracellular matrix protein ratios in the human heart and vessels: how to distinguish pathological from physiological changes? Front Physiol. (2021) 12:708656. doi: 10.3389/fphys.2021.708656 34421650 PMC8371527

[38] Chung WB Hamburg NM Holbrook M Shenouda SM Dohadwala MM Terry DF. The brachial artery remodels to maintain local shear stress despite the presence of cardiovascular risk factors. Arterioscler Thromb Vasc Biol. (2009) 29:606–12. doi: 10.1161/ATVBAHA.108.181495 PMC2719246 19164807

[39] Alhosseini Hamedani B Navidbakhsh M Ahmadi Tafti H. Comparison between mechanical properties of human saphenous vein and umbilical vein. BioMed Eng Online. (2012) 11:59. doi: 10.1186/1475-925X-11-59 22917177 PMC3527163

[40] Junek T Baum O Läuter H Vetter K Matejevic D Graf R. Pre-eclampsia associated alterations of the elastic fibre system in umbilical cord vessels. Anat Embryol (Berl). (2000) 201:291–303. doi: 10.1007/s004290050318 10794169

[41] Mochizuki S Brassart B Hinek A. Signaling pathways transduced through the elastin receptor facilitate proliferation of arterial smooth muscle cells. J Biol Chem. (2002) 277:44854–63. doi: 10.1074/jbc.M205630200 12244048

[42] Urbán Z Riazi S Seidl TL Katahira J Smoot LB Chitayat D. Connection between elastin haploinsufficiency and increased cell proliferation in patients with supravalvular aortic stenosis and Williams-Beuren syndrome. Am J Hum Genet. (2002) 71:30–44. doi: 10.1086/341035 12016585 PMC384991

[43] Lepedda AJ Nieddu G Formato M Baker MB Fernández-Pérez J Moroni L. Glycosaminoglycans: from vascular physiology to tissue engineering applications. Front Chem. (2021) 9:680836. doi: 10.3389/fchem.2021.680836 34084767 PMC8167061

[44] Mattson JM Turcotte R Zhang Y. Glycosaminoglycans contribute to extracellular matrix fiber recruitment and arterial wall mechanics. Biomech Model Mechanobiol. (2017) 16:213–25. doi: 10.1007/s10237-016-0811-4 PMC5288264 27491312

[45] Romanowicz L Sobolewski K. Extracellular matrix components of the wall of umbilical cord vein and their alterations in pre-eclampsia. J Perinat Med. (2000) 28:140–146. doi: 10.1515/JPM.2000.019 10875100

[46] Ghesquière L Perbet R Lacan L Hamoud Y Stichelbout M Sharma D. Associations between fetal heart rate variability and umbilical cord occlusions-induced neural injury: An experimental study in a fetal sheep model. Acta Obstet Gynecol Scand. (2022) 101:758–70. doi: 10.1111/aogs.14352 PMC9564451 35502642

[47] Maršál K. Physiological adaptation of the growth-restricted fetus. Best Pract Res Clin Obstet Gynaecol. (2018) 49:37–52. doi: 10.1016/j.bpobgyn.2018.02.006 29753694

[48] Driscoll AK Gregory ECW. Increases in prepregnancy obesity: United States, 2016–2019. In: NCHS Data Brief, no 392. National Center for Health Statistics, Hyattsville, MD (2020). 33270551

